# Development and external validation of a machine learning-based prognostic model for small cell neuroendocrine cervical carcinoma: a multi-center study

**DOI:** 10.1186/s12885-025-15338-8

**Published:** 2025-11-24

**Authors:** Yaxin Kang, Lele Chang, Huaqin Lin, Jing Liu, Cong Wang, Huaiwu Lu, Qin Xu

**Affiliations:** 1 Departments of Gynecology, Clinical Oncology School of Fujian Medical University, Fujian Cancer Hospital, NHC Key Laboratory of Cancer Metabolism, Fuzhou, Fujian 350014 China; 2 Department of Gynecologic Oncology, Shandong Cancer Hospital and Institute, Shandong First Medical University and Shandong Academy of Medical Sciences, Jinan, Shandong 250117 China; 3 Department of Gynecologic Oncology, Sun Yat-Sen Memorial Hospital, Guangzhou, Guangdong 510000 China; 4 Fujian Key Laboratory of Advanced Technology for Cancer Screening and Early Diagnosis, Fujian Cancer Hospital, Fuzhou, Fujian 350014 China

**Keywords:** Machine learning, Prognostic model, SCNECC, Multi-center study, SEER database

## Abstract

**Background:**

Small cell neuroendocrine cervical carcinoma (SCNECC) is a rare malignancy with a poor prognosis. The prognostic factors influencing SCNECC remain unclear. This study aimed to develop a prognostic model for SCNECC using machine learning (ML) techniques.

**Methods:**

We collected 487 patients diagnosed with SCNECC in the SEER database from 2004 to 2021, dividing them into a training set and an internal validation set at a 7:3 ratio. Additionally, we gathered 300 SCNECC patients from 3 Chinese registries between 2005 and 2023 as an external validation set. Initially, we performed univariate Cox regression analyses on 22 candidate variables using the Mime package. Variables with a *p*-value < 0.05 were included. Subsequently, to determine the optimal prognostic model, a total of 10 commonly used ML algorithms were collected and subsequently combined into 117 unique combinations. Finally, we validated the best model's performance using multiple independent cohorts, assessing metrics such as the concordance index (C-index), calibration curves, time-dependent receiver operating characteristic curves (ROC curves), and decision curve analyses (DCA).

**Results:**

The Stepwise Cox (StepCox) [forward] + Random Survival Forest (RSF) (SCR) model demonstrated the best predictive performance, with a C-index of 0.84 in the development set, 0.75 in the internal validation set, and 0.68 in the external validation set. It showed high prognostic value for 1-, 3-, and 5-year survival in SCNECC patients. SHAP-based interpretability analysis identified twenty key predictors that collectively enhanced the model's robustness.

**Conclusion:**

The SCR model has potential in predicting the prognosis of SCNECC, providing clinicians with decision support to identify high-risk patients, optimize treatment strategies, and ultimately improve clinical outcomes.

## Introduction

Small cell neuroendocrine carcinoma (SCNEC) is a unique and highly malignant pathological type that most commonly occurs in the lungs but can also arise in extra-pulmonary sites such as the endometrium and cervix [1–4]. Small cell neuroendocrine cervical carcinoma (SCNECC) is the most common neuroendocrine tumor in the female reproductive tract, but it is still relatively rare overall, accounting for only 0.5% to 5% of all cervical cancers. The incidence rate is approximately 0.06 per 100,000 [5]. It is strongly associated with high-risk human papillomavirus (HPV) infection and typically diagnosed in women aged 37–50 years [6–8]. Compared with squamous cell carcinoma or adenocarcinoma of the cervix, SCNECC demonstrates marked biological aggressiveness, frequent early distant metastasis, and dismal prognosis, with a median survival of less than one year in advanced stages [9–12].

Due to the rarity of SCNECC, there remains significant uncertainty regarding its recurrence patterns and prognostic risk factors [13]. Previous studies identified stage, age, and surgical status as potential prognostic factors and proposed simple nomogram models [14, 15]. However, these analyses were largely limited by small sample sizes, incomplete variable inclusion, and lack of robust external validation. A comprehensive, data-driven, and externally validated prognostic model for SCNECC is still lacking, underscoring the need for more sophisticated approaches to risk stratification.

In recent years, the rapid evolution of big-data analytics and high-performance computing has fostered the emergence of machine learning (ML) as a transformative methodology in medical research [16]. ML approaches can automatically identify complex, nonlinear relationships among multidimensional clinical variables, surpassing the capacity of traditional statistical models to capture hidden prognostic signals [17–19]. For rare malignancies such as SCNECC, machine learning enables the integration of large-scale heterogeneous datasets and provides a new avenue to develop robust, generalizable prognostic models that could meaningfully assist clinical decision-making [20].

In this study, we proposed a systematic multi-algorithm ML framework that integrates 10 mainstream survival-analysis methods into 117 unique hybrid model combinations to comprehensively explore the optimal predictive structure. Unlike prior single-cohort or single-algorithm studies, our model was developed on a large SEER-based population and externally validated across three independent Chinese tertiary hospitals, ensuring strong robustness and cross-regional generalizability. To our knowledge, this is the first ML–based, multi-center externally validated prognostic model for SCNECC. By combining a high-dimensional algorithmic ensemble with rigorous external validation, our work establishes a novel, reproducible, and clinically interpretable framework for individualized prognostic prediction in this rare and aggressive cervical malignancy. Integrating multiple ML algorithms would identify the optimal survival-prediction architecture for SCNECC and yield a robust, externally validated prognostic model capable of accurate individualized risk stratification across diverse populations.

## Materials and methods

To ensure the scientific rigor of the research design and the transparency of the results, this study strictly adhered to the Prediction model Risk Of Bias Assessment Tool (PROBAST), the criteria for the practicality of clinical prediction models proposed by Florian Markowetz, and the Transparent Reporting of a multivariable prediction model for Individual Prognosis Or Diagnosis (TRIPOD) guidelines [21–23].

### Patients

This study included two parts of data: (1) patients diagnosed with SCNECC from the Surveillance, Epidemiology, and End Results (SEER) database (2004–2021, United States), and (2) SCNECC patients from three tertiary hospitals in China (Fujian Cancer Hospital, Sun Yat-sen Memorial Hospital, and Shandong Cancer Hospital and Institute, 2005–2024).

The inclusion criterion was patients with pathologically confirmed SCNECC, and the exclusion criterion was patients with incomplete follow-up data. All enrolled patients signed informed consent, and the treatment process was guided by experienced clinicians. This study strictly followed the ethical principles established by the Declaration of Helsinki and was approved by the Ethics Committee of Fujian Cancer Hospital.

**Fig. 1:**
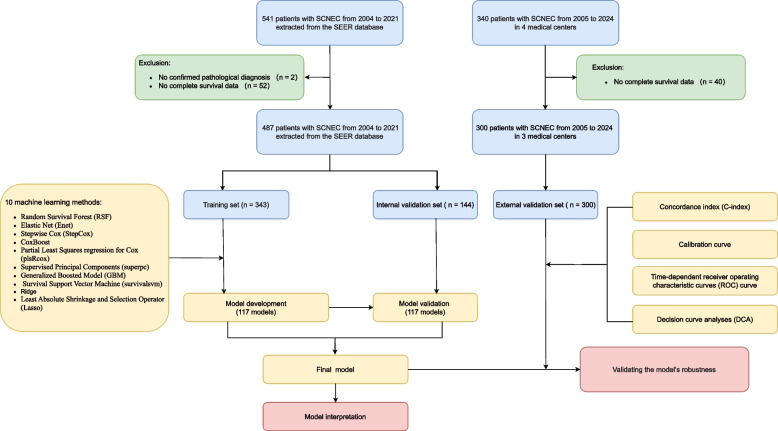
Study workflow and patient selection criteria

A total of 487 SEER patients were randomly divided into a training cohort (*n* = 343) and an internal validation cohort (*n* = 144) at a 7:3 ratio. An external validation cohort of 300 Chinese patients was used to independently assess model generalizability. The complete workflow of this study and the inclusion and exclusion criteria for patients were shown in Fig. 1.

### Data collection

In the training set, 22 clinical and pathological features were initially included as candidate predictive variables, such as diagnosis year, age, marital status, race, time from diagnosis to treatment, tumor size, first primary malignancy indicator, T stage, N stage, M stage, AJCC 8th stage, FIGO 2018th stage, combined stage, surgery, radiation prior to surgery, radiation after surgery, both surgery and radiation, radiation, chemotherapy, systemic therapy prior to surgery, systemic therapy after surgery, and both surgery and systemic therapy. To minimize coding and practice-related bias across centers, all variables were standardized before modeling, with harmonized definitions of staging (AJCC 8th/FIGO 2018th) and treatment modalities. Missing or unrecorded values were not imputed but were categorized as an “Unknown” level to retain all eligible cases and preserve statistical power.

### Model development and validation

To identify the optimal prognostic architecture, we implemented a multi-algorithm ML framework using the Mime R package (version 1.0.0; 10.1016/j.csbj.2024.2798; Liu H et al., 2024) [24]. Mime is a flexible and reproducible platform integrating multiple survival-analysis algorithms and supporting visualization of model construction and feature selection [25, 26].

Initially, univariate Cox regression was performed to select significant variables (*p* < 0.05) from the 22 candidates. The retained variables were entered into the ML framework comprising ten representative survival-analysis algorithms—Random Survival Forest (RSF), Elastic Net (Enet), Stepwise Cox (StepCox), CoxBoost, Partial Least Squares regression for Cox (plsRcox), Supervised Principal Components (superpc), Generalized Boosted Model (GBM), Survival Support Vector Machine (survivalsvm), Ridge, and Least Absolute Shrinkage and Selection Operator (LASSO). These algorithms were chosen to comprehensively represent the major paradigms of survival modeling—penalized regression, boosting, dimensionality reduction, tree-based ensemble learning, and traditional parametric approaches—thereby allowing the exploration of both linear and non-linear relationships within a unified machine-learning framework.

By systematically combining these algorithms and their tuned hyperparameters, a total of 117 predictive models were generated. Model performance was primarily assessed by the concordance index (C-index) through repeated cross-validation within the development cohort, while calibration curves, time-dependent receiver operating characteristic curves (ROC curves), and decision curve analyses (DCA) were used as complementary indicators. The model achieving the highest mean C-index with stable calibration across the training and internal validation cohorts was selected as the final prognostic model. Its reliability and applicability were subsequently confirmed in the independent external validation cohort, where individualized risk scores were calculated and patients were stratified into high- and low-risk groups.

### Statistical analyses

Categorical variables were expressed as frequencies (percentages). Comparisons between groups were performed using the chi-square test or Fisher's exact test. Continuous variables were expressed as medians (quartiles), and comparisons between groups were made using the rank sum test. The Kaplan–Meier curve was used to evaluate differences in OS between high and low-risk groups. All statistical tests were two-sided, and a *P*-value < 0.05 indicated statistical significance. All statistical analyses were performed using R version 4.3.2.

## Results

### Characteristics of patients

A total of 881 patients with SCNECC were included in this study. After applying the established inclusion and exclusion criteria, 94 patients who did not meet the conditions were excluded, leaving a final total of 787 patients for analyses. Table 1 summarized the baseline clinical characteristics of the training set (*n* = 343), internal validation set (*n* = 144), and external validation set (*n* = 300).

**Table 1:** Patient demographics and baseline characteristics from the training set, validation set, external validation set

Characteristic	Group			*p*-value^b^
	Train Set *N* = 343^a^	Val Set *N* = 144^a^	Ex Val Set *N* = 300^a^	
Year of diagnosis				< 0.001
2004–2010	100 (29.15%)	46 (31.94%)	32 (10.67%)	
2011–2016	124 (36.15%)	53 (36.81%)	82 (27.33%)	
2017 +	119 (34.69%)	45 (31.25%)	186 (62.00%)	
Age	49 (39, 61)	49 (37, 59)	48 (42, 54)	0.510
Marital status				< 0.001
Married	149 (43.44%)	57 (39.58%)	296 (98.67%)	
Others	194 (56.56%)	87 (60.42%)	4 (1.33%)	
Race				< 0.001
White	229 (66.76%)	104 (72.22%)	0 (0.00%)	
Black	63 (18.37%)	22 (15.28%)	0 (0.00%)	
Others	51 (14.87%)	18 (12.50%)	300 (100.00%)	
First malignant primary indicator				0.113
No	22 (6.41%)	9 (6.25%)	9 (3.00%)	
Yes	321 (93.59%)	135 (93.75%)	291 (97.00%)	
Time from diagnosis to treatment	26 (13, 42)	27 (11, 40)	13 (4, 25)	< 0.001
Tumor Size	63 (42, 80)	60 (34, 80)	46 (32, 60)	< 0.001
T				< 0.001
T1	109 (31.78%)	57 (39.58%)	77 (25.67%)	
T2	93 (27.11%)	31 (21.53%)	153 (51.00%)	
T3	72 (20.99%)	36 (25.00%)	25 (8.33%)	
T4	20 (5.83%)	3 (2.08%)	9 (3.00%)	
Unknown	49 (14.29%)	17 (11.81%)	36 (12.00%)	
N				< 0.001
N0	129 (37.61%)	54 (37.50%)	163 (54.33%)	
N1	187 (54.52%)	76 (52.78%)	108 (36.00%)	
Unknown	27 (7.87%)	14 (9.72%)	29 (9.67%)	
M				< 0.001
M0	202 (58.89%)	91 (63.19%)	234 (78.00%)	
M1	134 (39.07%)	49 (34.03%)	38 (12.67%)	
Unknown	7 (2.04%)	4 (2.78%)	28 (9.33%)	
AJCC 8th Stage				< 0.001
Stage Ⅰ	67 (19.53%)	38 (26.39%)	76 (25.33%)	
Stage Ⅱ	32 (9.33%)	9 (6.25%)	134 (44.67%)	
Stage Ⅲ	84 (24.49%)	40 (27.78%)	20 (6.67%)	
Stage Ⅳ	141 (41.11%)	49 (34.03%)	40 (13.33%)	
Unknown	19 (5.54%)	8 (5.56%)	30 (10.00%)	
FIGO 2018th Stage				< 0.001
Stage Ⅰ	64 (18.66%)	33 (22.92%)	53 (17.67%)	
Stage Ⅱ	27 (7.87%)	8 (5.56%)	84 (28.00%)	
Stage Ⅲ	117 (34.11%)	60 (41.67%)	93 (31.00%)	
Stage Ⅳ	122 (35.57%)	36 (25.00%)	42 (14.00%)	
Unknown	13 (3.79%)	7 (4.86%)	28 (9.33%)	
Combined Stage				< 0.001
Localized	60 (17.49%)	33 (22.92%)	151 (50.33%)	
Regional	130 (37.90%)	57 (39.58%)	83 (27.67%)	
Distant	141 (41.11%)	49 (34.03%)	38 (12.67%)	
Unknown	12 (3.50%)	5 (3.47%)	28 (9.33%)	
Surgery				< 0.001
No	235 (68.51%)	93 (64.58%)	117 (39.00%)	
Yes	108 (31.49%)	51 (35.42%)	183 (61.00%)	
Radiation prior to surgery				< 0.001
No/Unknown	97 (28.28%)	46 (31.94%)	160 (53.33%)	
Yes	11 (3.21%)	5 (3.47%)	23 (7.67%)	
No surgery	235 (68.51%)	93 (64.58%)	117 (39.00%)	
Radiation after surgery				< 0.001
No/Unknown	53 (15.45%)	27 (18.75%)	78 (26.00%)	
Yes	55 (16.03%)	24 (16.67%)	105 (35.00%)	
No surgery	235 (68.51%)	93 (64.58%)	117 (39.00%)	
Both surgery and radiation				
No	279 (81.34%)	115 (79.86%)	173 (57.67%)	
Yes	64 (18.66%)	29 (20.14%)	107 (35.67%)	
Unknown	0 (0.00%)	0 (0.00%)	20 (6.67%)	
Radiation				0.230
No	121 (35.28%)	62 (43.06%)	119 (39.67%)	
Yes	222 (64.72%)	82 (56.94%)	181 (60.33%)	
Chemotherapy				0.156
No	50 (14.58%)	25 (17.36%)	61 (20.33%)	
Yes	293 (85.42%)	119 (82.64%)	239 (79.67%)	
Systemic therapy prior surgery				< 0.001
No/Unknown	84 (24.49%)	44 (30.56%)	114 (38.00%)	
Yes	24 (7.00%)	7 (4.86%)	69 (23.00%)	
No surgery	235 (68.51%)	93 (64.58%)	117 (39.00%)	
Systemic therapy after surgery				< 0.001
No/Unknown	32 (9.33%)	18 (12.50%)	11 (3.67%)	
Yes	76 (22.16%)	33 (22.92%)	172 (57.33%)	
No surgery	235 (68.51%)	93 (64.58%)	117 (39.00%)	
Both surgery and systemic therapy				
No	241 (70.26%)	101 (70.14%)	126 (42.00%)	
Yes	91 (26.53%)	37 (25.69%)	174 (58.00%)	
Unknown	11 (3.21%)	6 (4.17%)	0 (0.00%)	
OS				< 0.001
Lived	117 (34.11%)	51 (35.42%)	173 (57.67%)	
Death	226 (65.89%)	93 (64.58%)	127 (42.33%)	

^a^n (%); Median (Q1, Q3)

^b^Pearson's Chi-squared test; Kruskal–Wallis rank sum test

In the training set, the median age of the patients was 49 years (IQR: 39–61), with 43.44% being married and 66.76% being White. Most patients were in the advanced stages (Stage III–IV, 65.60%), and the proportion of patients subjected to surgical treatment was 31.49%. By the end of follow-up, 65.89% of patients had experienced a fatal event.

In the internal validation set, the median age of patients was also 49 years (IQR: 37–59). Married patients accounted for 39.58%, and the proportion of White was 72.22%. The proportion of patients in the advanced stages was 61.81%, the surgical treatment rate was 35.42%, and the mortality rate was 64.58%.

In the external validation set, the median age of patients was 48 years (IQR: 42–54). Compared to the other cohorts, this cohort had a lower proportion of patients in the advanced stages, which was only 20.00%. However, the proportion of patients subjected to surgical treatment was higher (61.00%). The mortality rate was 42.33%, which was significantly lower than that of the training and internal validation set.

### Model development

Among the 22 initially collected candidate clinical and pathological variables, a univariate Cox proportional hazards analysis was conducted to identify those significantly associated with overall survival (OS). Two variables—radiation prior to surgery and systemic therapy prior to surgery—did not show statistically significant associations with OS (*p* > 0.05) and were therefore excluded before model construction. This exclusion step was implemented to minimize potential noise, prevent overfitting, and ensure that only clinically relevant and statistically validated predictors were included in the subsequent modeling process. Finally, we leaved the following 20 variables for inclusion in the ML modeling process: age, race, time from diagnosis to treatment, T stage, N stage, M stage, AJCC 8th stage, FIGO 2018th stage, combined stage, surgery, radiation prior to surgery, radiation after surgery, both surgery and radiotherapy, radiation, chemotherapy, systemic therapy before surgery, systemic therapy after surgery, both surgery and systemic therapy, tumor size, and first malignant primary indicator.

**Fig. 2:**
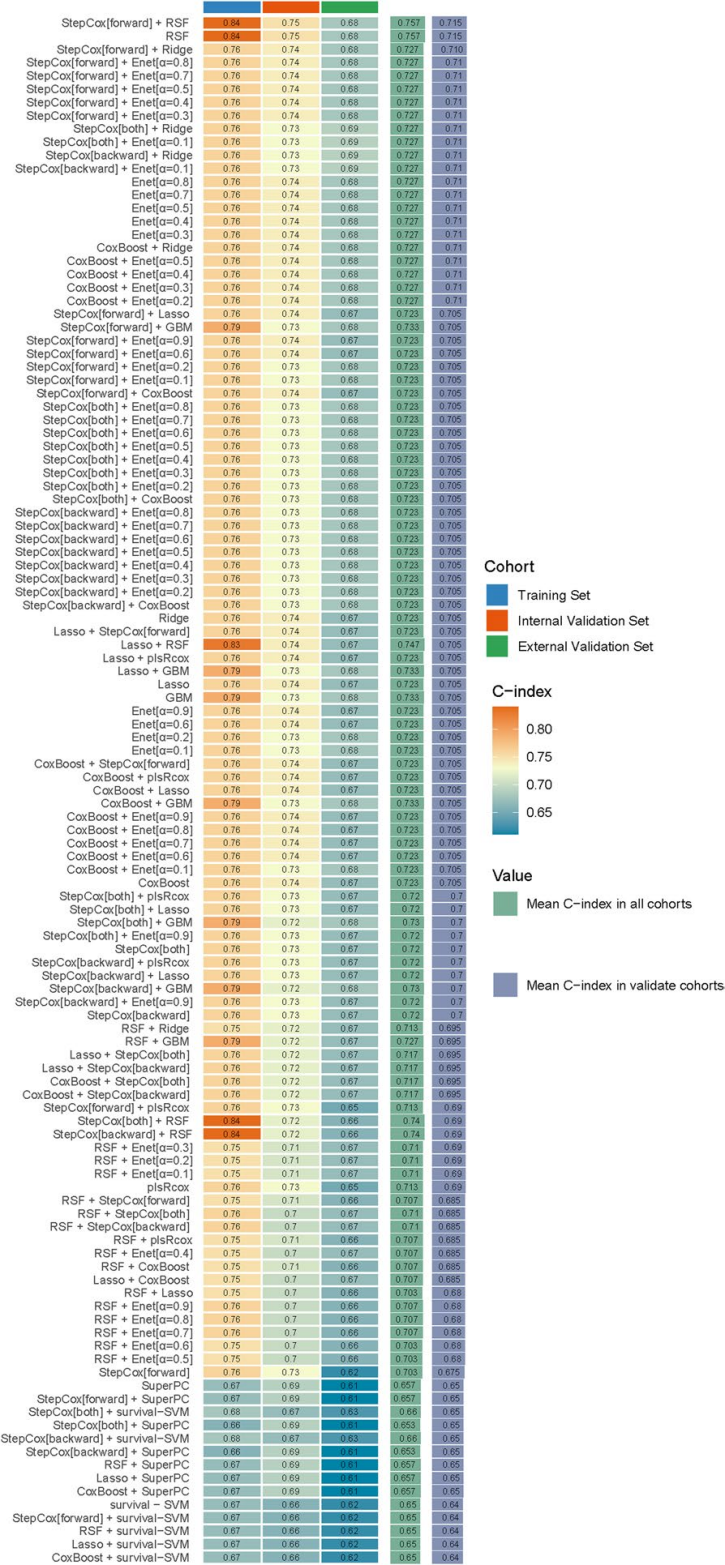
Model performance comparison based on concordance index (C-index)

Using the "Mime" package, a systematic arrangement of combinations of the aforementioned variables was conducted across 10 mainstream ML algorithms, resulting in the construction of 117 predictive models. Subsequently, model performance evaluations were carried out in two independent validation cohorts. Fig. 2 illustrated the C-index performance of each model in different datasets. Among all 117 candidate combinations, the model constructed by combining StepCox (forward method) with RSF—denoted as the StepCox [forward] + RSF (SCR) model achieved the highest mean C-index across both the internal and external validation cohorts (0.750 in the internal validation set, and 0.680 in the external validation set), indicating superior generalizability and stability compared with other algorithms. The SCR model integrates a linear feature-selection layer with a non-linear ensemble learning stage. Specifically, the StepCox component identifies stable and statistically significant predictors through forward selection, effectively reducing dimensionality and collinearity. The selected variables are then input into the RSF module, which models higher-order interactions and complex non-linear effects that conventional Cox regression cannot capture. This hybrid design combines interpretability with predictive flexibility, allowing robust identification of key prognostic factors while improving discrimination and calibration performance in heterogeneous clinical datasets.

### Model validation

**Fig. 3:**
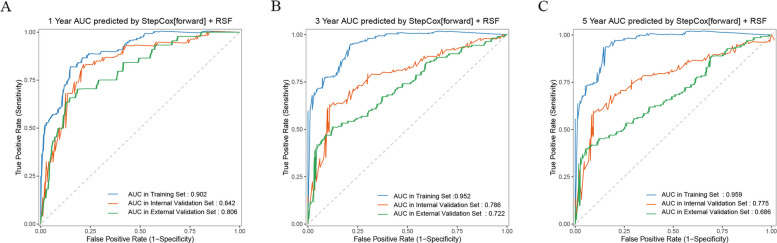
Calibration curves of the SCR model for 1-, 3-, and 5-year overall survival. **A** Development set, (**B**) internal validation set, and (**C**) external validation set

**Fig. 4:**
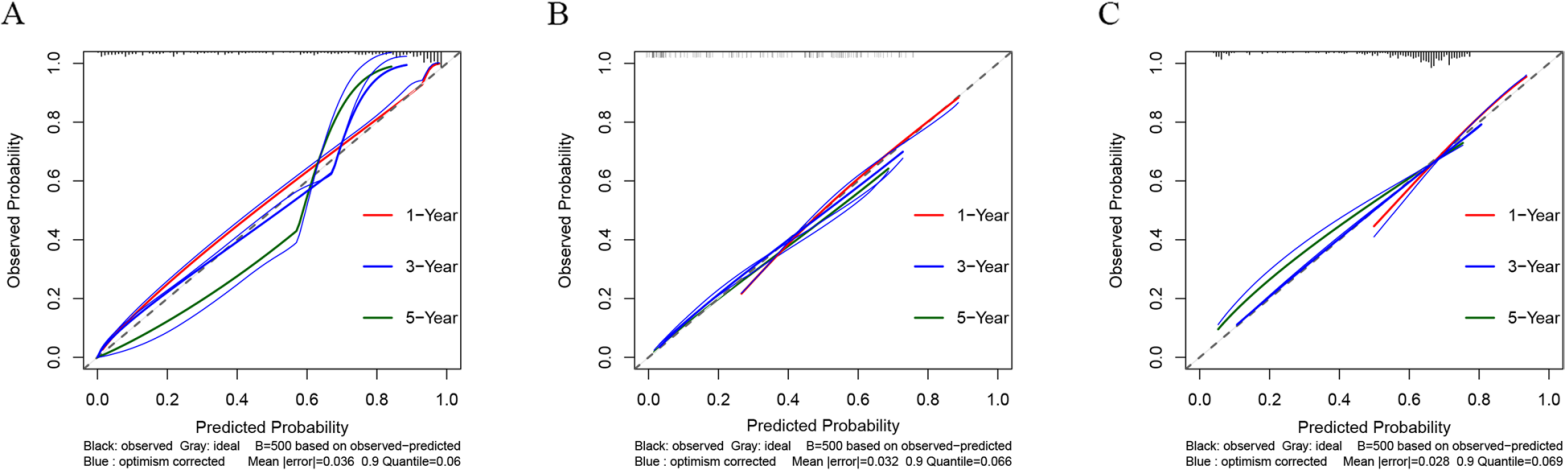
Time-dependent receiver operating characteristic (ROC) curves of the SCR model

The SCR model demonstrated good fitness in calibration curves at 1-, 3-, and 5-year, achieving reasonable calibration performance in both the development set and the two validation cohorts (Fig. 3). Simultaneously, the time-dependent ROC curve further verified the strong predictive ability of the SCR model for survival outcomes at different time points. In the development set, the AUCs of the model for 1-, 3-, and 5-year survival rates were 0.902, 0.952, and 0.959, respectively; in the internal validation set, they were 0.842, 0.786, and 0.775; and 0.806, 0.722, and 0.686 in the external validation set (Fig. 4), showing good time prediction performance.

**Fig. 5:**
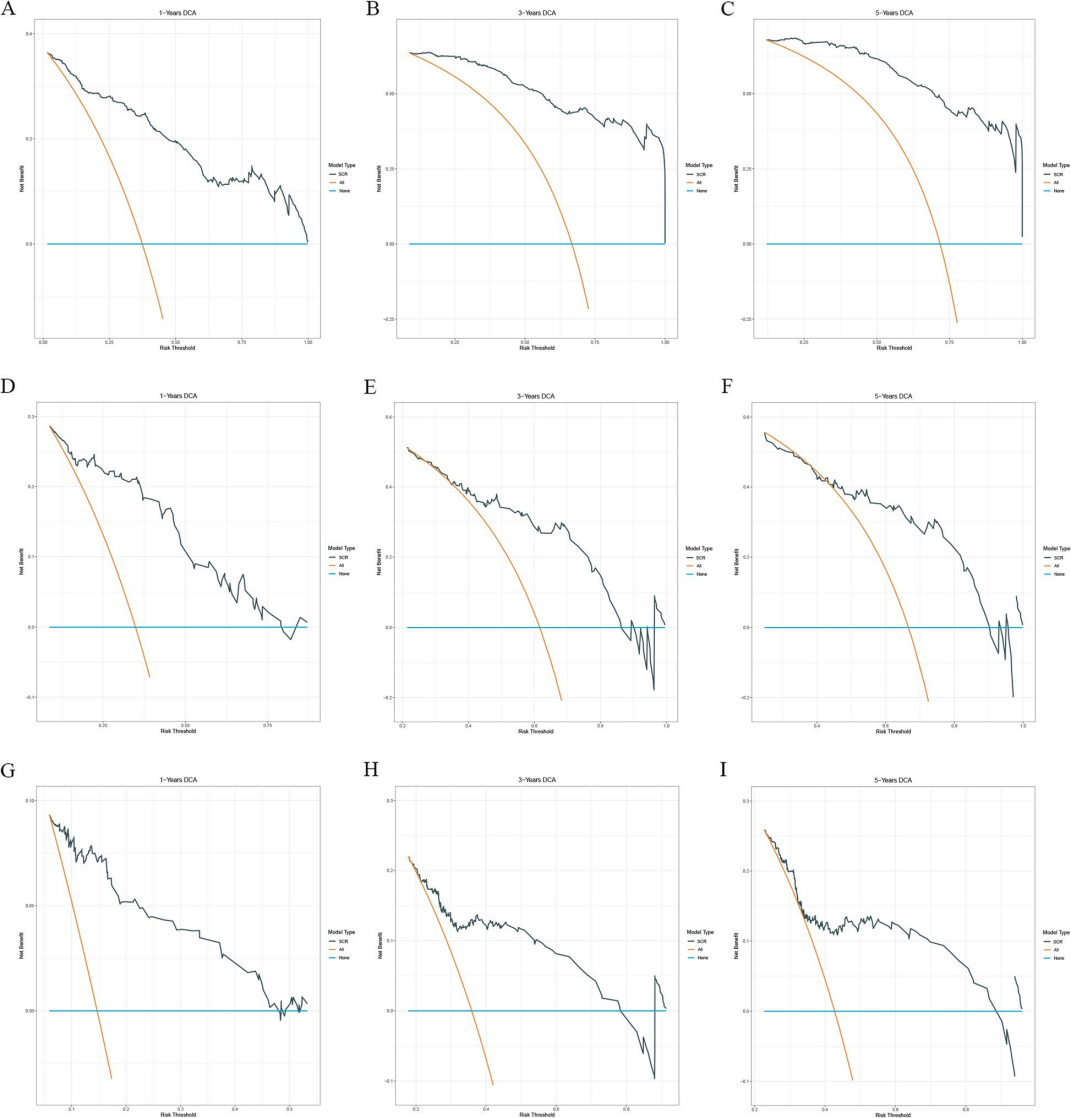
Decision curve analysis (DCA) of the SCR model for clinical utility. Net benefit curves for 1-, 3-, and 5-year survival predictions in (**A**) development, (**B**) internal validation, and (**C**) external validation cohorts

The results of DCA indicated that the SCR model had high clinical net benefit in predicting 1-, 3-, and 5-year survival in all three cohorts, suggesting its strong potential for application in practical clinical decision-making (Fig. 5).

**Fig. 6:**
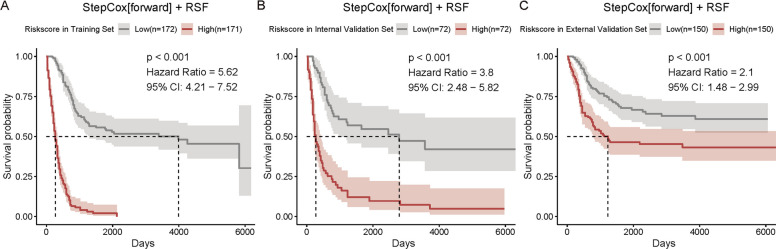
Survival analysis stratified by SCR model risk groups. Kaplan–Meier curves comparing overall survival between high-risk and low-risk groups (stratified by median risk score) in (**A**) development, (**B**) internal validation, and (**C**) external validation cohorts

Additionally, individualized risk scores were calculated for each patient based on the SCR model, and patients were subsequently stratified into high-risk and low-risk groups according to the median cutoff of predicted risk scores. Survival analyses demonstrated significant differences in OS between the two groups across all cohorts (Fig. 6). Patients classified as high-risk exhibited markedly poorer prognoses compared with the low-risk group, with hazard ratios (HRs) of 5.62 (95% CI: 4.21–7.52) in the training cohort, 3.80 (95% CI: 2.48–5.82) in the internal validation cohort, and 2.10 (95% CI: 1.48–2.99) in the external validation cohort (*p* < 0.001 for all).

### Model explained

**Fig. 7:**
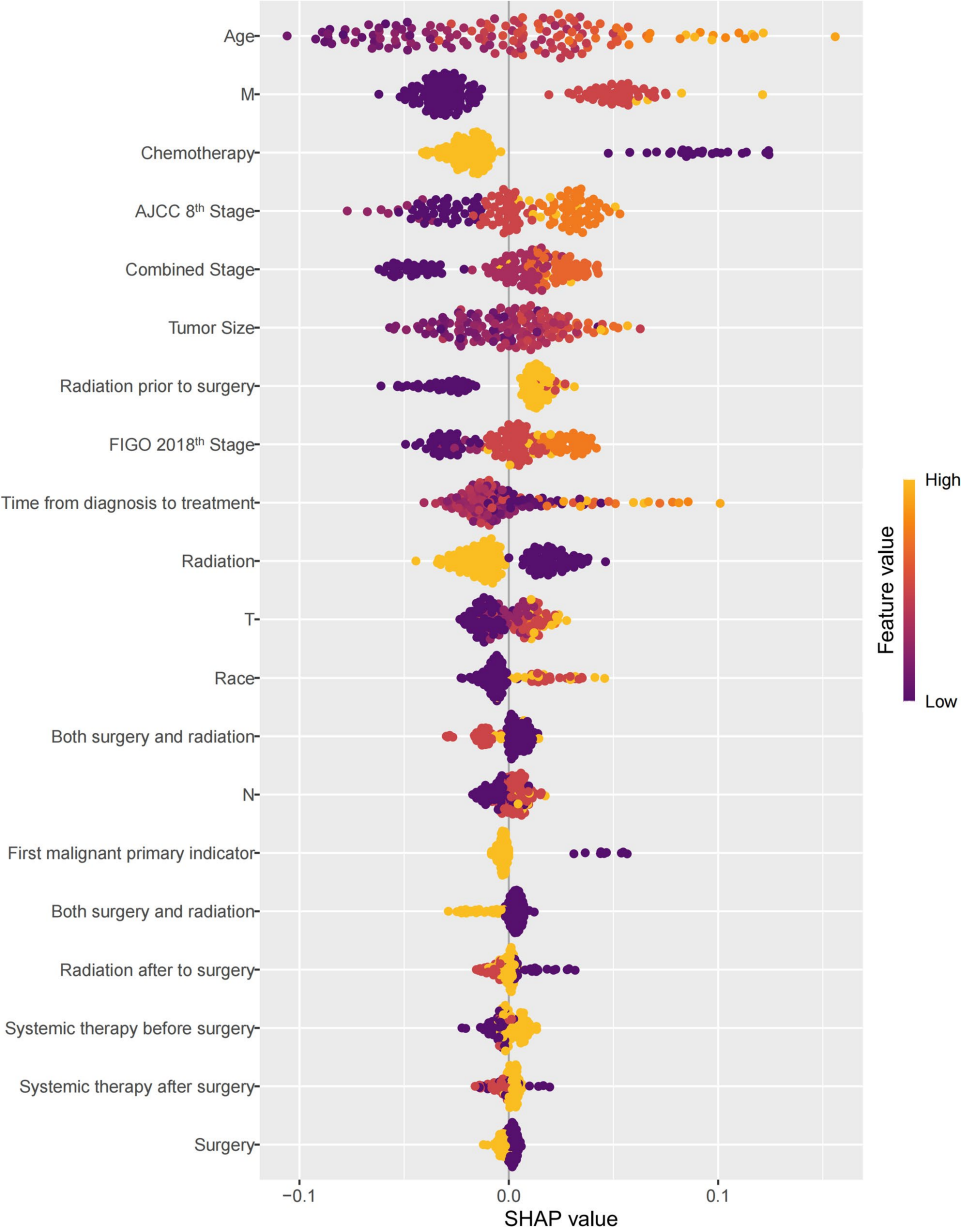
SHAP summary plot demonstrating feature importance and directional impact on predicted overall survival in the SCR model

The SHapley Additive exPlanations (SHAP) summary plot illustrates the contribution and directionality of each variable to the predicted overall survival in the SCR model. Chemotherapy, M stage, and age were identified as the most influential predictors, followed by combined stage, tumor size, and FIGO 2018th stage. Patients with higher M stage, advanced clinical stage, larger tumor size, or older age tended to have positive SHAP values, suggesting a higher predicted mortality risk. Conversely, receipt of chemotherapy, radiotherapy, and surgical intervention yielded negative SHAP values, implying protective effects and improved survival (Fig. 7).

## Discussion

This study developed and validated a multi-algorithm ML model, termed SCR, to predict the OS of patients with SCNECC. To our knowledge, this is the first ML-based, multi-center externally validated prognostic model specifically designed for SCNECC. The model integrated 10 classical survival algorithms and employed rigorous internal and external validation, which enhanced its generalizability and reproducibility.

Because SCNECC exhibits highly heterogeneous biological behavior and complex interdependencies among clinicopathological factors, traditional single-form statistical models (e.g., Cox regression or nomogram) were insufficient to capture these non-linear prognostic patterns. In contrast, our multi-algorithm machine-learning framework systematically benchmarked 117 model combinations—including penalized Cox variants, tree-based learners, and boosting architectures—and identified the SCR model as the most robust structure [15, 27]. This hybrid design effectively balanced interpretability with non-linear modeling capacity, yielding superior external generalizability across heterogeneous cohorts (Fig. 2). This methodological innovation supported more nuanced risk stratification and provides a foundation for data-driven clinical decision support in this rare malignancy.

Our analyses revealed notable differences between the SEER and Chinese multicenter cohorts. The proportion of FIGO stage IV patients in the SEER cohort (32.44%) was substantially higher than in the Chinese cohort (14.00%), partially explaining the superior survival observed in the latter. In the SCR model, surgery was included as a key input variable after univariate screening and consistently ranked among the top contributors in the RSF-based variable-importance analysis. Patients who received curative surgical treatment predominantly belonged to the lower-risk group defined by the model, underscoring its prognostic relevance and its central role in refining individualized risk stratification. These findings align with existing clinical evidence and highlight the importance of timely surgical management in SCNECC whenever feasible [9, 28].

To further elucidate the clinical interpretability of the SCR model, we performed a SHAP analysis in the internal validation cohort (Fig. 7). This approach quantifies the individualized contribution and directionality of each variable to the survival prediction, thereby providing a more intuitive understanding of how clinical factors shape the model’s risk estimation. The SHAP plot identified FIGO 2018th stage, tumor size, surgery, and age as the most influential predictors of overall survival. Patients with advanced FIGO 2018th stages (III–IV) and larger tumors exhibited positive SHAP values, indicating strong contributions to increased mortality risk, which aligns with the well-recognized aggressiveness and metastatic potential of high-stage SCNECC. Conversely, surgery demonstrated prominent negative SHAP values, reflecting its protective effect. This observation corroborates clinical evidence that definitive surgery, when feasible, substantially improves survival outcomes even in SCNECC [6]. Older patients showed higher SHAP values and thus higher predicted mortality risk, consistent with decreased treatment tolerance and higher comorbidity burden. Collectively, the SHAP analysis complements traditional variable-importance results by visualizing both the magnitude and direction of each variable’s impact on individualized predictions. These clinically interpretable patterns reinforce the reliability of the SCR model and highlight modifiable treatment-related factors—particularly surgery and adjuvant therapy—as critical determinants of improved prognosis in SCNECC.

According to the predicted risk scores derived from the SCR model, patients were dichotomized into high- and low-risk groups using the median cutoff. Those assigned to the high-risk group were more likely to be older, have distant metastases, and not receive chemotherapy—features that align closely with established indicators of poor prognosis [29]. Survival analyses further confirmed that high-risk patients had significantly poorer OS compared with low-risk patients across all datasets (p < 0.001). This integrated analysis not only enhances the biological and clinical interpretability of the SCR model but also provides a data-driven, clinically relevant framework for identifying SCNECC patients at elevated mortality risk.

Moreover, the SCR model exhibited consistent performance across heterogeneous populations, including SEER and multiple Chinese validation cohorts, despite differences in demographics, disease stage, and treatment modalities. Notably, the C-index declined from 0.840 in the development cohort to 0.680 in the external validation cohort, primarily due to marked heterogeneity between the SEER and Chinese datasets. The external cohort showed a lower mortality rate and longer OS—with many patients surviving beyond 6000 days—as well as a higher proportion of early- to mid-stage disease and greater surgical intervention. These differences in case-mix, event rate, and follow-up likely narrowed the risk distribution and reduced model discrimination. Despite this, the SCR model consistently achieved significant survival separation across all datasets, indicating robust generalizability while highlighting the need for cohort-specific recalibration in future applications.

From a biological perspective, previous molecular studies have suggested that persistent HPV infection and aberrant transcriptional regulation drive neuroendocrine differentiation and the aggressive phenotype of SCNECC [30]. These mechanisms may partly underlie the high recurrence rate and poor prognosis observed clinically. In the future, with the deepening of basic research, accumulation of clinical data, and the introduction of technologies such as artificial intelligence big models, we hope to further enhance our understanding of the pathophysiology and molecular characteristics of SCNECC, thereby providing patients with more precise diagnosis and treatment strategies and ultimately improving their clinical outcomes.

Despite the promising results of this study, several limitations should be acknowledged. Firstly, the SCR model was built based on the SEER database, and differences in racial backgrounds and healthcare systems may affect its applicability to other populations. Secondly, the model did not incorporate key variables such as pathological features and detailed treatment information, which may have restricted the further improvement of its predictive ability. Additionally, patients with incomplete follow-up were excluded to ensure the accuracy of survival outcomes, which may introduce minor selection bias. Although multi-center external validation helped mitigate this limitation. Finally, the retrospective nature of the study introduces potential selection and information biases. Looking ahead, we plan to improve the predictive accuracy of the SCR model through several strategies, including the integration of multi-modal data (radiomic, molecular, and pathological features), exploration of advanced ensemble and deep learning architectures, and prospective recalibration across larger, more diverse populations.

## Conclusion

In conclusion, this study established and externally validated a multi-algorithm ML framework for prognostic modeling in SCNECC. The SCR model demonstrated reasonable discrimination, calibration, and generalization performance in both internal and external cohorts. While its predictive accuracy remains moderate, the model provides a preliminary, data-driven approach for individualized risk stratification and hypothesis generation. With further prospective validation and integration of radiomic, molecular, and clinical parameters, this framework may ultimately contribute to more precise management of patients with SCNECC.

## Data Availability

The data supporting this study’s findings are available from the corresponding author upon reasonable request.
